# Understanding the patterns and predictors of elevated psychological distress among humanitarian migrants compared to the host population: comparative matched analysis using two national data sources from Australia

**DOI:** 10.1017/S2045796025100139

**Published:** 2025-07-07

**Authors:** Demelash Woldeyohannes Handiso, Jacqueline A. Boyle, Eldho Paul, Frances Shawyer, Graham Meadows, Joanne C. Enticott

**Affiliations:** 1 Monash Centre for Health Research and Implementation, Monash University, Australia; 2 Health Systems and Equity, Eastern Health Clinical School, Department of Obstetrics and Gynaecology, Monash University, Australia; 3 Department of Psychiatry, Southern Synergy, Monash University, Melbourne, VIC, Australia; 4 School of Primary and Allied HealthCare, Monash University, Melbourne, VIC, Australia; 5 Centre for Mental Health and Community Wellbeing, School of Population and Global Health, University of Melbourne, Melbourne, VIC, Australia

**Keywords:** Australian-born, Australian National Health Survey, Building a New Life in Australia, elevated psychological distress, humanitarian migrants

## Abstract

**Aims:**

Understanding patterns and predictors of elevated psychological distress (EPD) among humanitarian migrants compared to the host population is critical for designing effective mental health interventions. However, existing research presents conflicting findings on the prevalence of EPD. This study examined EPD prevalence and associated factors in humanitarian migrants and Australian-born adults using large population-level datasets.

**Methods:**

Kessler 6 scores (range 6–30) were dichotomised, and scores above 19 were defined as EPD and indicative of probable serious mental illness. Comparative 1:2 matched analysis used humanitarian migrant data from the Building a New Life in Australia and Australian-born comparators from the National Health Survey. Each humanitarian migrant was matched by age, sex and location with two Australian-born residents. Modified Poisson regression identified predictors of EPD in both groups.

**Results:**

EPD was higher among humanitarian migrants (17.2%, 95% CI: 15.5, 18.9) compared to Australian-born (14.5%, 95% CI: 13.3, 15.6), with an adjusted relative risk (aRR) with 95% confidence intervals (1.16%, 95% CI: 1.11, 1.21) after adjusting for key factors. In both groups, females had a higher aRR than males, with similar effect sizes: 1.06 (95% CI: 1.04, 1.08) among Australian-born and 1.04 (95% CI: 1.02, 1.07) among humanitarian migrants. The impact of age on distress was more pronounced in Australian-born individuals: compared to the 65+ age group, the youngest group (18–24 years) had an aRR of 1.36 (95% CI: 1.28, 1.43) for Australian-born and 1.19 (95% CI: 1.12, 1.27) for humanitarian migrants. Compared to excellent health, poor and fair self-rated health condition had an aRR of 2.13 (95% CI: 2.03, 2.26) and 1.69 (95% CI: 1.61, 1.79), respectively, for humanitarian migrants and 1.94 (95% CI: 1.82, 2.05) and 1.48 (95% CI: 1.43, 1.56), respectively, for Australian born. Australian-born individuals in the lowest-income quintile had higher distress (aRR: 1.11 [95% CI: 1.06–1.15]) compared to the highest-income quintile, with no significant income effect for humanitarian migrants. In both groups, females with poorer self-rated health had higher aRRs than females reporting excellent health.

**Conclusions:**

Although distress prevalence was higher in the humanitarian migrants, age and sex differences followed similar patterns in both groups. Income level was a factor in Australian-born adults but not in humanitarian migrants. Clinically, this highlights the need for culturally sensitive and group-specific mental health support. From a policy perspective, the use of matching methodology from large, separate datasets offers a valuable model for generating actionable insights, supporting the development of targeted and equitable mental health programmes.

## Introduction

According to the United Nations High Commissioner for Refugees (UNHCR), the number of forcibly displaced individuals reached 110 million by mid-2023, marking a 1% rise from the end of 2022 (UNHCR, 2024). In response to evolving global needs since World War II, Australia’shumanitarian programme has expanded to offer permanent resettlement opportunities, with 17,875 placements offered in 2022–2023 and an intake of 20,000 places planned for both 2023–2024 and 2024–2025 (Australian Government Department of Home Affairs, 2024; Karlsen *et al.*, 2010). Humanitarian migrants are displaced persons who have been granted protection in the resettled country or have been resettled through programmes outside the asylum procedure (Bîrgău, 2022).

Humanitarian migrants often face numerous challenges during and after their migration process, including trauma, persecution, and difficulties establishing a new life in the countries of resettlement (Australian Government Department of Home Affairs, 2023; Jenkinson *et al.*, 2016). These hardships are not unique to Australia; similar experiences are observed in other high-income host nations (Sigvardsdotter *et al.*, 2016). Many humanitarian migrants are at increased risk of mental health issues due to their past experiences and ongoing stressors, such as limited access to mental health services (Handiso *et al.*, 2024; Sarría-Santamera *et al.*, 2016; Silver *et al.*, 2023), discrimination, lack of social support (Alegría *et al.*, 2017; Handiso *et al.*, 2024) and financial hardship (Handiso *et al.*, 2024; Schenker, 2010). Furthermore, humanitarian migrants may be more vulnerable to elevated psychological distress (EPD) than host populations due to unique and compounding risk factors such as family separation, cultural shock, language barriers and social exclusion, which intensify post-migration stress and hinder social integration (Jurado *et al.*, 2017; Miller *et al.*, 2018; Naseh *et al.*, 2024; Nguyen *et al.*, 2024). EPD, characterised by emotional suffering with symptoms such as depression and anxiety, is common among humanitarian migrants and often exacerbated by the compounding effects of pre- and post-migration adversities (Keramat *et al.*, 2024; Mirowsky and Ross, 2002).

However, research comparing the mental health status of humanitarian migrants with that of host populations has produced conflicting findings. Some studies show higher levels of EPD among humanitarian migrants, while others indicate higher levels among the host population or no significant differences (Giacco *et al.*, 2018; Priebe *et al.*, 2016). For instance, some studies have reported EPD of over 20–35%% among humanitarian migrants (Walther *et al.*, 2020), while others have found similar or even higher rates among host populations, ranging from 20% to 35%, highlighting the inconsistency in findings and the need for further comparative research (Giuliani *et al.*, 2023; Lindert *et al.*, 2009). Methodological limitations such as non-comparable datasets, small or non-representative samples, inconsistent mental health measures and limited use of matching methods reduce the utility of these findings for policymakers and programme planners (Minas *et al.*, 2013; Watson and Wooden, 2002).

This study addressed these gaps by employing a high-quality dataset, advanced matching approaches, a validated data collection tool and a representative sample from both the Australian-born group and humanitarian migrants, to compare EPD. We aimed to provide robust evidence on the patterns and predictors of EPD in these two groups and to explore whether demographic, social and socio-economic factors account for any observed disparities. By doing so, we hope to inform more targeted and effective mental health interventions and policies for humanitarian migrants.

## Methods

### Data source and samples

Our study used two data sets to compare EPD among humanitarian migrants and Australian-born adults: humanitarian migrants’ data were from the fifth wave of the Building a New Life in Australia (BNLA) survey conducted in 2017/18, and the nationally representative Australian National Health Survey (ANHS) conducted in 2017/18. Using data sources from the same year allowed us to compare the humanitarian migrants and Australian-born populations directly. The response rates for the Australian-born database and the humanitarian migrant’s database were approximately 76.1% and 81.0%, respectively (Australian Bureau of Statistics, 2018; Australian Government: Department of Social Services, 2022).

The BNLA dataset was a longitudinal survey of 2,399 humanitarian migrants. It provides a nationally representative sample of individuals from diverse cultural backgrounds. These participants arrived in Australia and received permanent protection visas through the country’s humanitarian programmes between May and December 2013 (Australian Government: Department of Social Services, 2022). Data collectors obtained a list of all eligible participants from the Australian Government’s Department of Immigration and Border Protection, and study participants provided consent to participate in the survey before data collection commenced. The survey tool was checked for internal consistency and translated into 19 languages to ensure that most participants could respond in their primary language (Australian Government: Department of Social Services, 2022).

**Figure 1.**
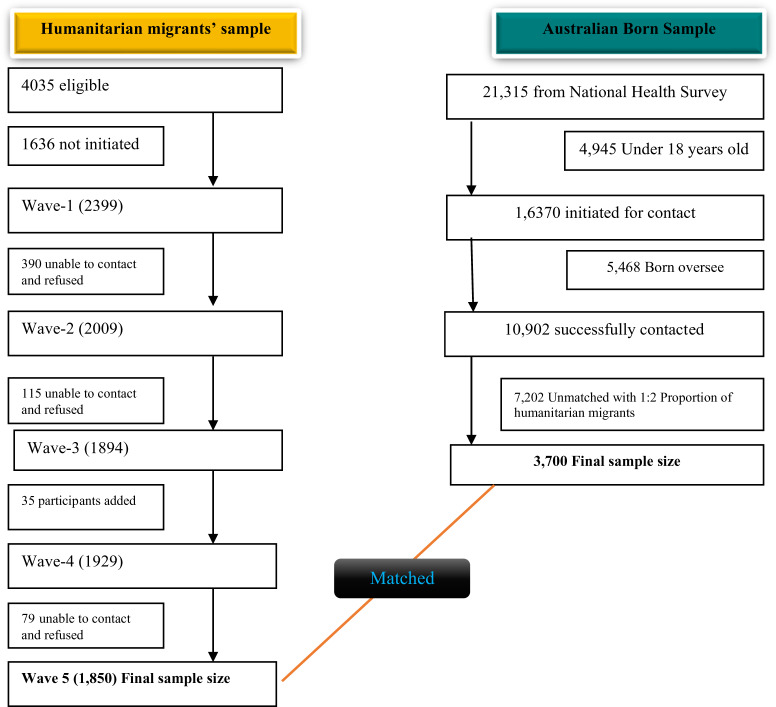
Flow chart for selecting and matching study participants from humanitarian migrants and the Australian-born population.

The ANHS was an ongoing national health survey designed to collect a comprehensive range of information about the health of the Australian population, including data on the prevalence of various health conditions and mental well-being. The 2018 survey data were accessed through the public use microdata file platform, and then a sample consisting solely of Australian-born individuals were extracted for the matched analysis (Australian Bureau of Statistics, 2021). This comparison group was used to avoid mixing Australian-born individuals with other migrants. The sample included a 1:2 ratio of humanitarian migrants and Australian-born participants. To maintain this proportional representation, we randomly selected two Australian-born participants for every one humanitarian migrant included in the analysis. Matching variables were selected based on relevance to outcome variables and their availability and comparability across both datasets, comprising age, gender and remoteness/location (Figure 1).

### Outcome measures

The ANHS reported the EPD ratings for each of the 10 items in the 10-item Kessler Psychological Distress scale (K10) (Kessler *et al.*, 2002). However, the BNLA dataset had only six of these same items, called the 6-item Kessler scale (K6). Therefore, we examined the K6 items in both surveys. K6 scale scores (Australian scoring) range from 6 to 30. The Australian Bureau of Statistics (ABS) recommends, in dichotomous analysis, the use of a score of 19 or higher to define EPD (Kessler *et al.*, 2010) and is indicative of probable serious mental illness (Australian Bureau of Statistics, 2021). These scores (≥19) will here be described as ‘EPD’ (Australian Bureau of Statistics, 2012; Harvard University, 2010).

### Independent variables

The following covariates were investigated in both datasets. Sex was asked as a binary of male and female. Age by years was categorised into the following groups: 18–24 years, 25–34 years, 35–44 years, 45–54 years, 55–64 years and 65+ years. Educational status was asked as highest level completed then classified according to the International Standard Classification of Education (ISCED) (Australian Bureau of Statistics, 2015) as High education ([Levels 5–8]: ISCED 5: Short-cycle tertiary education; ISCED 6: Bachelor’s or equivalent level; ISCED 7: Master’s or equivalent level; ISCED 8: Doctoral or equivalent level); Medium education ([Levels 3 and 4]: ISCED 3: Upper secondary education; ISCED 4: Post-secondary non-tertiary education); and Low education ([Levels 0–2]: ISCED 0: Early childhood education [‘less than primary’ for educational attainment]; ISCED 1: Primary education; ISCED 2: Lower secondary education). Self-rated health condition (excellent, very good, good, fair, poor), income Quintile (Quintile 1–5) and employment included full-time/part-time/casual (yes/no).

Two area covariates were also investigated: (1) Location/remoteness was classified as major cities, inner regional, and outer/other regional based on the Australian Bureau of Statistics (21), and (2) Index of Relative Socio-economic Disadvantage (IRSD) (Quintile 1–5), also determined by the ABS using a variety of area-based information such that IRSD Quintile 1 represents the most disadvantaged areas.

### Statistical analyses

Statistical analysis was performed using Stata version 18 (StataCorp, 2023) to investigate the relationship between EPD and the characteristics of humanitarian migrants and Australian-born. A modified Poisson regression model with robust variance was used to identify predictors of EPD for both groups. Univariable and multivariable analyses were conducted to determine the associations between covariates and outcome variables. Crude and adjusted relative risks (aRR) with 95% confidence intervals were presented for predictor variables with *P*-values <0.05. The variable selection process for the final model employed adaptive Least Absolute Shrinkage and Selection Operator (LASSO) methods. Cross-validation was used to determine the optimal alpha hyperparameter to ensure the best performance of the LASSO regression. The final model included variables such as self-rated health condition, location/remoteness, employment status, Index of Relative Socio-economic Disadvantage (IRSD) for the area, income, educational status, age categories and sex. The Hosmer–Lemeshow statistic and omnibus tests assessed the goodness of fit. We tested interaction effects for the outcome variables; however, a significant interaction was observed only between sex and self-rated health in the final step of the model. Additionally, we estimated marginal effects to measure changes in the likelihood of EPD given self-rated health conditions, age, income level, and IRSD quintile for both humanitarian migrants and the Australian-born. We followed the STrengthening the Reporting of OBservational studies in Epidemiology (STROBE) (Cooper *et al.*, 2019) guidelines in reporting this study. Missing data were handled using multiple imputation by chained equations (MICE), following StataCorp’s recommendations. This process involved constructing imputation models for all explanatory variables with missing values. The Stata commands ‘mi impute’ and ‘mi estimate’ were then used to generate, combine and analyse data across 20 imputed datasets (StataCorp, 2023).

## Results

### Characteristics of study participants

The main analysis included 5,550 participants, comprising 1,850 humanitarian migrants and 3,700 matched (by age, sex and location/remoteness) individuals born in Australia. Table 1 displays the characteristics of the humanitarian migrant and Australian-born study participants across various socio-demographic, educational and socio-economic variables. In both samples, most participants were male (52.6%) and aged between 18 and 44 (64.1%). Most of the female (69.2%) and male (64.1%) humanitarian migrants had low education levels, whereas the largest proportion of Australian-born women had a high level of education (46.9%), and in men, the largest proportion had a medium level of education (47.7%). Employment status differs significantly between humanitarian migrants and the Australian-born group. Most humanitarian migrants resided in the most disadvantaged IRSD areas, 61.1% (n = 1131), compared to the Australian-born counter sample; household income showed a similar trend (Table 1).

**Table 1.** Socio-demographic characteristics of Australian-born and humanitarian migrant study participants, *n* (%)

		Humanitarian migrants (1850)		Australian-born (3700)	
Variables	Categories	Male (974)	Female (876)	Male (1948)	Female (1752)
Age (years)	18−24	139 (14.3)	135 (15.4)	278 (14.3)	270 (15.4)
	25−34	232 (23.8)	228 (26.0)	464 (23.8)	456 (26.0)
	35−44	237 (24.3)	214 (24.4)	474 (24.3)	428 (24.4)
	45−54	202 (20.7)	165 (18.8)	404 (17.5)	330 (18.8)
	55−64	95 (9.8)	77 (8.8)	190 (9.8)	154 (8.8)
	65+	69 (7%)	57(6.6)	138 (7%)	114 (6.6)
Educational status	Low education	617 (64.1)	602 (69.2)	297 (15.3)	261 (14.9)
	Medium education	177 (18.3)	154 (17.7)	930 (47.7)	669 (38.2)
	High education	169 (17.6)	114 (13.1)	721 (37.0)	822 (46.9)
Location	Major cities	901 (92.5)	807 (92.1)	1802 (92.5)	1614 (92.1)
	Inner regional	56 (5.8)	65 (7.4)	112 (5.8)	130 (7.4)
	Outer/other regional	17 (1.8)	4 (0.5)	34 (1.8)	8 (0.5)
Employment	Yes	83 (8.6)	7 (0.8)	1876 (96.3)	1691 (96.5)
	No	884 (91.4)	859 (99.2)	72 (3.7)	61 (3.5)
Self-rated health condition	Excellent	168 (17.2)	104 (11.9)	365 (18.7)	357 (20.4)
	Very good	209 (21.5)	155 (17.7)	732 (37.6)	671 (38.3)
	Good	259 (26.6)	216 (24.7)	575 (29.5)	507 (28.9)
	Fair	183 (18.8)	206 (23.5)	215 (11.0)	168 (9.6)
	Poor	155 (15.9)	195 (22.3)	61 (3.1)	49 (2.8)
IRSD area quintile	Quintile 1	595 (61.3)	536 (61.6)	250 (12.8)	240 (13.7)
	Quintile 2	180 (18.5)	152 (17.5)	344 (17.7)	299 (17.1)
	Quintile 3	110 (11.3)	97 (11.6)	414 (21.3)	378 (21.6)
	Quintile 4	61 (6.3)	63 (7.2)	464 (23.8)	417 (23.8)
	Quintile 5	25 (2.6)	22 (2.5)	476 (24.4)	418 (23.9)
Income quintile	Quintile 1	585 (60.1)	511 (58.2)	225 (11.6)	253 (14.4)
	Quintile 2	133 (13.7)	115 (13.1)	248 (12.7)	271 (15.5)
	Quintile 3	179 (18.4)	182 (20.9)	411 (21.1)	339 (19.4)
	Quintile 4	48 (4.9)	40 (4.7)	467 (23.9)	431 (24.6)
	Quintile 5	29 (9)	28 (3.1)	597 (30.7)	458 (26.1)
Disability	Yes	104 (10.9)	78 (9.1)	414 (21.3)	384 (21.9)
	No	845 (89.1)	775 (90.9)	1534 (78.8)	1368 (78.1)

IRSD: Index of Relative Socio-economic Disadvantage for areas, IRSD Quintile 1 represents the most disadvantaged areas.

### Comparison of EPD between humanitarian migrants and Australian-born

**Figure 2.**
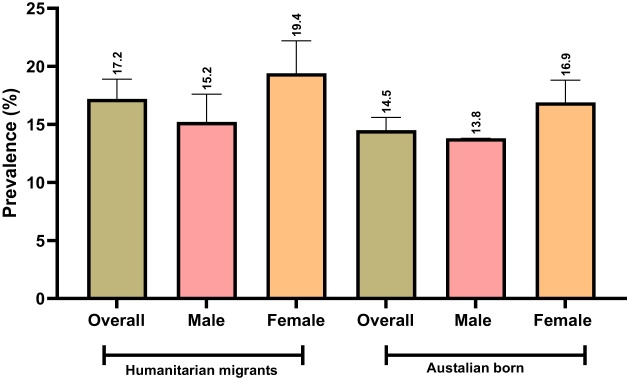
Prevalence of EPD in humanitarian migrants and matched Australian-born study participants (95% confidence interval).

The overall prevalence of EPD was higher among humanitarian migrants (17.2%, 95% CI: 15.5, 18.9) compared to Australian-born adults (14.5%, 95% CI: 13.3, 15.6), and this occurred for both females and males (Fig. 2).

A higher proportion of Australian-born females (23.3%) and males (16.2%) in the youngest age group (18–24) reported EPD compared with 8.9% and 9.4% of female and male humanitarian migrants, respectively. For individuals with low educational status, EPD was observed in 14.6% (*n* = 90) male and 19.3% (*n* = 116) female humanitarian migrants, compared to 22.6% (*n* = 67) male and 23.4% (*n* = 61) female individuals in the Australian-born. A greater proportion of Australian-born adults reported EPD if they self-reported fair or poor health. For those with fair health, EPD levels were 8.8% and 20.7% higher in Australian-born males and females, respectively, compared to humanitarian migrants. For those with poor health, EPD was 11.9% higher in males and 19.2% higher in females among the Australian-born population (Table 2).

**Table 2.** Prevalence of EPD between humanitarian migrants and Australian-born, stratified by gender across various factors

		Humanitarian migrants			Australian-born		
Variables		Male, yes *n* (%)	Female, yes *n* (%)	*P*-value	Male, yes *n* (%)	Female, yes *n* (%)	*P*-value
Age (years)	18−24	13 (9.4)	12 (8.9)	<0.001	45 (16.2)	63 (23.3)	<0.001
	25−34	27 (11.6)	25 (10.9)		59 (12.7)	77 (16.9)	
	35−44	36 (15.2)	39 (18.2)		46 (9.7)	68 (15.9)	
	45−54	45 (22.3)	56 (33.9)		51 (12.6)	55 (16.7)	
	55−64	17 (17.9)	23 (29.9)		17 (8.9)	25 (16.2)	
	65+	10 (14.5)	15 (26.3)		20 (14.5)	9 (7.8)	
Educational status	Low	90 (14.6)	116 (19.3)	<0.001	67 (22.6)	61 (23.4)	<0.001
	Medium	25 (14.1)	31 (20.1)		109 (11.7)	135 (20.3)	
	High	28 (16.6)	22 (19.3)		62 (8.6)	101 (12.3)	
Location	Major	139 (15.4)	159 (19.7)	0.0039	220 (12.2)	271 (16.8)	<0.001
	Inner	7 (12.5)	11 (16.9)		17 (15.2)	23 (17.7)	
	Outer/other	2 (11.8)	0		1 (2.9)	3 (37.5)	
Employment status	Yes	6 (7.2)	1 (14.3)	<0.001	222 (11.8)	276 (16.3)	<0.001
	No	139 (15.7)	165 (19.2)		16 (22.2)	21 (34.4)	
Self-rated health condition	Excellent	4 (2.4)	3 (2.9)	<0.001	14 (3.8)	25 (7.0)	<0.001
	Very	8 (3.8)	4 (2.6)		52 (7.1)	78 (11.6)	
	Good	23 (8.9)	27 (12.5)		70 (12.2)	92 (18.2)	
	Fair	40 (21.9)	42 (20.4)		66 (30.7)	69 (41.1)	
	Poor	73 (47.1)	94 (48.2)		36 (59.0)	33 (67.4)	
IRSD	Quintile 1	96 (16.1)	112 (20.9)	<0.001	53 (21.2)	64 (26.7)	0.008
	Quintile 2	29 (16.1)	24 (15.8)		44 (12.8)	58 (19.4)	
	Quintile 3	12 (10.9)	20 (20.6)		47 (11.4)	53 (14.0)	
	Quintile 4	7 (11.5)	9 (14.3)		58 (12.5)	81 (19.4)	
	Quintile 5	3 (12.0)	3 (13.6)		36 (7.6)	41 (9.8)	
Income quintile	Quintile 1	98 (16.8)	105 (20.8)	0.113	66 (29.3)	69 (27.3)	< 0.001
	Quintile 2	15 (11.3)	18 (15.4)		40 (16.1)	62 (22.9)	
	Quintile 3	27 (15.1)	35 (19.2)		44 (10.7)	52 (15.3)	
	Quintile 4	6 (12.5)	8 (20.0)		51 (10.9)	52 (12.1)	
	Quintile 5	2 (6.9)	4 (10.7)		37 (6.2)	62 (13.5)	

IRSD: Index of Relative Socio-economic Disadvantage for areas; IRSD Quintile 1 represents the most disadvantaged areas.

Humanitarian migrants residing in IRSD Quintile 1 reported EPD in 16.1% (*n* = 96) males and 20.9% (*n* = 112) females. In comparison, the Australian-born showed distress in 21.2% (*n* = 53) males and 26.7% (*n* = 64) females, indicating a 5.1% and 5.8% higher distress in Australian-born males and females, respectively. In Quintile 2, EPD was 3.3% higher in humanitarian migrant males, while it was 3.6% higher in females from the Australian-born (Table 2).

### Predictors of EPD in humanitarian migrant and Australian-born

After adjusting for socio-demographic factors, self-rated health condition, gender and age category emerged as key determinants of EPD in both Australian-born individuals and humanitarian migrants (Table 3). However, remoteness, income and IRSD area quintiles were determinant factors exclusively for the Australian-born population.

**Table 3.** Crude relative risk estimates in comparing EPD between humanitarian migrants and a matched Australian-born group

Variables	Variable categories	Australian-born cRR (95% CI)	Humanitarian migrants cRR (95% CI)
Sex **(Male #**)	Female	1.07 (1.04, 1.09)	1.11 (1.08, 1.14)
Age category **(65+ years #)**	18−24 years	1.21 (1.15, 1.27)	0.82 (0.77, 0.87)
	25−34 years	1.14 (1.09, 1.20)	0.85 (0.80, 0.89)
	35−44 years	1.11 (1.05, 1.16)	0.92 (0.87, 0.97)
	45−54 years	1.10 (1.05, 1.16)	1.02 (0.97, 1.08)
	55−64 years	1.04 (0.98, 1.09)	1.08 (1.01, 1.14)
Educational status **(Low education#)**	Medium	0.93 (0.90, 0.96)	1.01 (0.97, 1.04)
	High	0.88 (0.86, 0.92)	1.01 (0.97, 1.05)
Income quintile **(Quintile 5#)**	Quintile 1	1.23 (1.18, 1.27)	1.09 (1.01, 1.17)
	Quintile 2	1.12 (1.08, 1.17)	0.96 (0.89, 1.05)
	Quintile 3	1.04 (1.01, 1.08)	1.11 (1.03, 1.20)
	Quintile 4	1.02 (0.99, 1.05)	0.99 (0.89, 1.09)
IRSD **(Quintile 5#)**	Quintile 1	1.17(1.12, 1.21)	1.08 (0.98, 1.17)
	Quintile 2	1.09 (1.05, 1.13)	1.00 (0.92, 1.09)
	Quintile 3	1.05 (1.01, 1.08)	0.99 (0.91, 1.09)
	Quintile 4	1.08 (1.05, 1.12)	0.95 (0.86,1.04)
Employment **(Yes#)**	No	1.18 (1.11, 1.24)	1.25 (1.17, 1.33)
Location **(Outer regional#)**	Major cities	1.14 (1.02, 1.28)	1.13 (0.99, 1.29)
	Inner region	1.15 (1.02, 1.29)	1.00 (0.87, 1.15)
Self-rated health condition **(Excellent#)**	Very good	1.12 (1.09, 1.16)	1.17 (1.11, 1.24)
	Good	1.21 (1.17, 1.25)	1.34 (1.27, 1.40)
	Fair	1.49 (1.44, 1.56)	1.66 (1.58, 1.74)
	Poor	1.92 (1.81, 2.03)	2.08 (1.99, 2.18)
Both matches **(Australian-born#)**	Humanitarian migrants		1.49 (1.46, 1.51)

cRR: Crude Relative Risk; IRSD: Index of Relative Socio-economic Disadvantage for areas; IRSD Quintile 1 represents the most disadvantaged areas.

**Figure 3.**
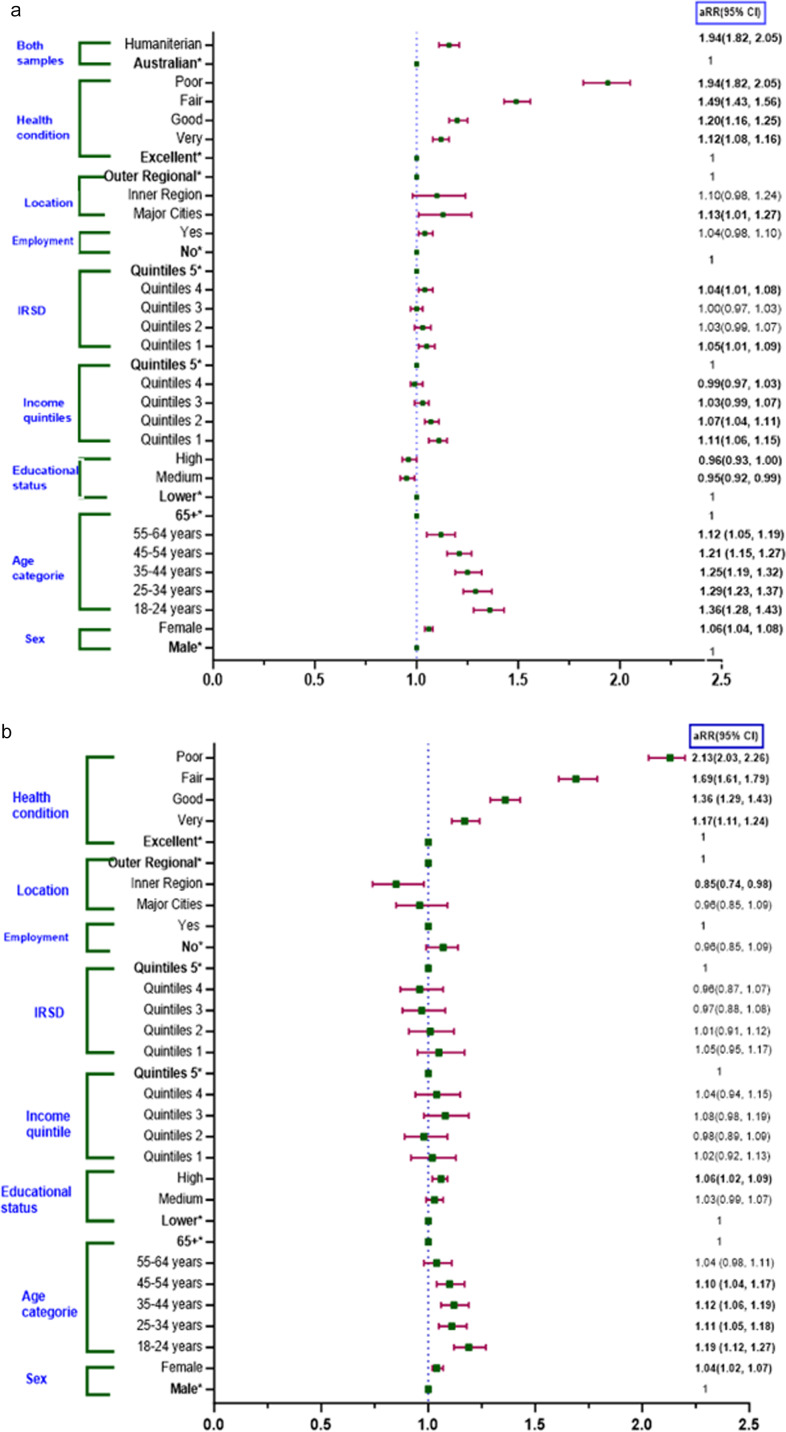
Relative risk estimates comparing EPD between Australian-born (a) and a matched humanitarian migrants (b). IRSD: index of relative socio-economic disadvantage for areas (IRSD Quintile 1 represents the most disadvantaged areas); aRR: adjusted risk ratio (adjusted for self-rated health, age, sex, employment status and location). Note: * indicates the reference categories.

**Figure 4.**
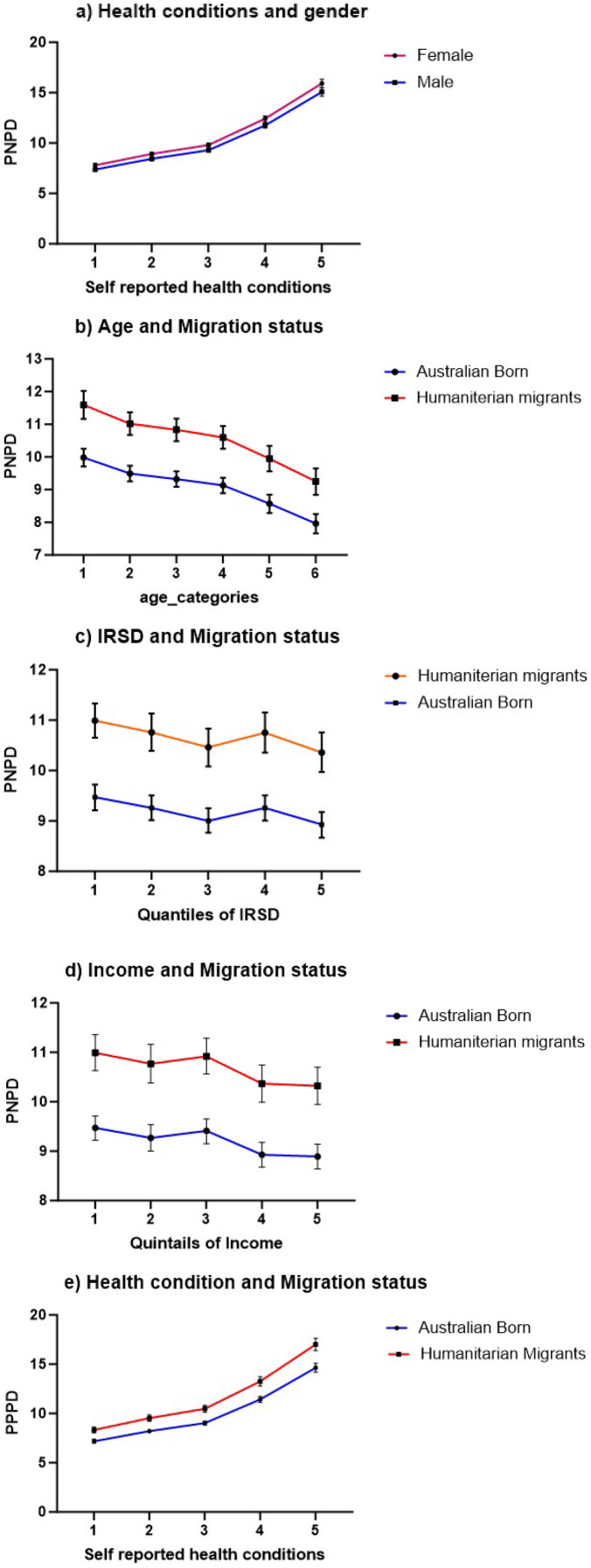
Marginal effects on EPD score of humanitarian migrants and Australian-born. (a) Margin effect of gender and self-rated health conditions; (b) margin effect between age and migration status; (c) margin effect of IRSD quintile and migration status; (d) margin effect of income level and migration status; (e) margin effect of self-rated health condition and migration status.

Overall, humanitarian migrants experienced a 16% higher relative risk of EPD compared to Australian-born individuals (aRR: 1.16, 95% CI: 1.11–1.21). In both the Australian-born and humanitarian migrants, females had a greater relative risk of distress compared to males, and the magnitude of this effect appeared similar in the two groups: females exhibited a 6% increased relative risk of EPD compared to males among Australian-born individuals (aRR: 1.06, 95% CI: 1.04–1.08) and 4% higher relative risk among humanitarian migrants (aRR: 1.04, 95% CI: 1.02–1.07). However, the distress effect size for age was more significant in the Australian-born compared to humanitarian migrants, as compared to the 65+ age group, the youngest group of 18–24 years had a relative risk of 1.36 for Australian-born and 1.19 for humanitarian migrants (aRR: 1.36; 95% CI: 1.28–1.43) and (aRR: 1.19; 95% CI: 1.12–1.27) respectively. Australian-born individuals, those aged 25–34 and 35–44, have a 1.29 (aRR: 1.29; 95% CI: 1.23–1.37) and 1.25 (aRR: 1.25; 95% CI: 1.19–1.32) times higher relative risk, respectively, whereas humanitarian migrants in these same age groups have a comparatively lower relative risk, with aRRs of 1.11 (95% CI: 1.05–1.18) for ages 25–34 and 1.12 (95% CI: 1.06–1.19) for ages 35–44, relative to those over 65 (Fig. 3). However, the marginal effect indicates that humanitarian migrants consistently experience higher levels of EPD than Australian-born individuals across all age categories. Although distress levels decrease slightly with age, the gap between the two groups remains significant (Fig. 4).

In an Australian-born study, participants with lower income quintiles were associated with an increased relative risk of EPD compared to the fifth quintile, particularly in Quintiles 1 and 2, which had 1.11- and 1.07-times higher relative risk of EPD, respectively (aRR: 1.11; 95% CI: 1.06–1.15 and aRR: 1.07; 95% CI: 1.04–1.11). Australian-born individuals residing in IRSD Quintile 1 had a 5% higher relative risk, and those in Quintile 4 had a 4% higher relative risk of EPD compared to those in the Quintile 5 (aRR: 1.05; 95% CI: 1.01–1.09 and aRR: 1.04; 95% CI: 1.01–1.08). For Australian-born individuals, medium levels of education were associated with a 5% lower relative risk of EPD compared to lower education levels (aRR: 0.95; 95% CI: 0.92–0.99). In contrast, humanitarian migrants with higher education face a 6% higher relative risk of EPD compared to those with lower education levels (aRR: 1.06; 95% CI: 1.02–1.09). Among Australian-born individuals, those living in major cities were at 1.13 times higher relative risk of EPD compared to those residing in outer regions (aRR: 1.13; 95% CI: 1.01–1.27) (Fig. 3).

The effect size for EPD was higher among humanitarian migrants than Australian-born individuals, particularly when self-rated health was considered. Humanitarian migrants who rated their health as poor and fair had a 2.13- and 1.69-times higher relative risk of experiencing EPD, respectively, compared to those reporting excellent health (aRR: 2.13; 95% CI: 2.03–2.26 and aRR: 1.69; 95% CI: 1.61–1.79) (Fig. 3). Marginal effects further indicate that humanitarian migrants consistently experience higher EPD across various health conditions (Fig. 4).

Additionally, we examined the relative risk of EPD by considering the interaction between self-rated health and gender for both Australian-born individuals and humanitarian migrants. In both groups, poorer self-rated health was associated with higher aRR across genders. For Australian-born individuals, males with poor health have an aRR of 1.93, while females have an aRR of 1.94. Humanitarian migrants show even higher aRRs, with males at 2.09 and females at 2.20. Humanitarian migrants experience higher aRRs than Australian-born individuals (Table 4).

**Table 4.** Interaction term for self-rated health condition and gender in Australian and humanitarian migrants

		Australian-born aRR (95% CI)	Humanitarian migrants aRR (95% CI)
**Interaction term**	Excellent*Female		
Self-rated health condition * Gender	Very good*Male	1.12 (1.08, 1.18)	1.13 (1.05, 1.21)
	Very good*Female	1.11 (1.06, 1.17)	1.25 (1.15, 1.35)
	Good*Male	1.19 (1.13, 1.25)	1.35 (1.27, 1.44)
	Good*Female	1.21 (1.16, 1.28)	1.38 (1.28, 1.49)
	Fair*Male	1.46 (1.38, 1.55)	1.66 (1.55, 1.78)
	Fair*Female	1.53 (1.44, 1.63)	1.76 (1.63, 1.89)
	Poor*Male	1.93 (1.78, 2.08)	2.09 (1.96, 2.25)
	Poor*Female	1.94 (1.79, 2.12)	2.20 (2.04, 2.38)

## Discussion

The study revealed that humanitarian migrants (17.2%) experienced higher levels of EPD compared to Australian-born adults (14.5%), with a 1.16 times higher relative risk (aRR) after adjusting for factors such as self-rated health condition, age, gender, employment status and location. In both groups, females exhibited a higher risk of EPD than males, with comparable effect sizes. However, age played a more substantial role in EPD among Australian-born individuals, with younger adults demonstrating higher aRRs than older adults. Poor and fair self-rated health conditions had a higher impact on distress among humanitarian migrants compared to Australian-born individuals. Additionally, among Australian-born individuals, those in the lowest income quintiles exhibited significantly higher distress risks than those in the highest income quintile, while no significant income-related effects were observed for humanitarian migrants.

Younger study participants and women in both groups exhibited more EPD compared to older participants and males; this observation aligns with a study from the United States and another study conducted in Australia (Jarallah and Baxter, 2019; Li and Anderson, 2016). The higher distress experienced by migrants in the younger age group may be at least partially attributable to stresses associated with displacement, including difficulties in acclimating to a newly resettled country, social isolation, pressure to adapt rapidly to a new culture and identity struggles (Cooper *et al.*, 2019; Guskovict and Potocky, 2018; Handiso *et al.*, 2024). The increased distress observed in women and younger persons from both groups may be ascribed to their heightened susceptibility (Yuan *et al.*, 2009). Among Australian-born individuals, the relative risk of EPD was lower with moderate education relative to lower education levels, consistent with established knowledge, as elevated educational attainment is often associated with enhanced access to resources, superior socio-economic status, and more effective coping mechanisms (Ouwehand *et al.*, 2009; Roohafza *et al.*, 2009). Education may offer a sense of agency and stability, mitigating the effects of stressors (Brännlund and Hammarström, 2014; Mandemakers and Monden, 2010). In terms of self-assessed health, Australians in poor health had the highest levels of EPD relative to humanitarian migrants, perhaps attributable to elevated expectations and perceived inequities in healthcare access (Brijnath *et al.*, 2020).

Lower income level was also correlated with increased EPD in both groups. Low-income individuals face financial hardship as well as limited access to healthcare, which can exacerbate EPD (Fukuda and Hiyoshi, 2012; Isaacs *et al.*, 2018). Nevertheless, Australian-born individuals in lower-income quintiles exhibited higher EPD than humanitarian migrants, possibly due to greater expectations and perceived disparities in socio-economic status and access to resources (Brijnath *et al.*, 2020). Although the host population often have more stable living conditions, access to mental health services varies substantially with socio-economic disadvantage in the general population (Dawadi *et al.*, 2024). In some settings, humanitarian and non-governmental support may enhance service availability for migrants (Steel *et al.*, 2009). As well, humanitarian migrants may have developed resilience from overcoming past adversities, which might buffer against EPD despite lower socio-economic conditions (Majumder,^2016^).

Australian-born adults with fair or poor self-rated health conditions had higher EPD than humanitarian migrants. These discrepancies could result from better healthcare access in the host population, leading to higher detection and disclosure of EPD (Selkirk *et al.*, 2014). In contrast, humanitarian migrants face barriers to accessing healthcare services, stigma and cultural differences, which have been found to result in lower reported distress levels (Jarallah and Baxter, 2019; Priebe *et al.*, 2016). The host population may also have more chronic health conditions due to prolonged exposure to risk factors, contributing to a higher risk of EPD (Debnar *et al.*, 2020).

Finally, EPD in Australian-born decreased with age. The oldest age group (65+) and those aged 55–64 had lower relative risk compared to younger groups, supported by other studies in Australia (Jorm *et al.*, 2005) but contrasting with others (Kilkkinen *et al.*, 2007). Younger people experience more transitions, social pressures and uncertainties, contributing to higher distress levels than older individuals (Matud *et al.*, 2020).

### Policy implication

Humanitarian migrants experience 16% higher EPD compared to the Australian-born population, making targeted mental health services and support systems essential, including early identification using screening and diagnostic tools in clinical visits (Shawyer *et al.*, 2017, 2014). Policymakers should ensure these services are accessible to this group. Implementing community-based mental health programmes, improving access to culturally familiar support systems, and expanding transcultural mental health services can further enhance outcomes. In addition, addressing the identified disparities in education between humanitarian migrants and Australians is critical. Programmes promoting educational attainment and vocational training can empower individuals, alleviate financial stress and reduce EPD. For Australian-born individuals, particularly those in lower-income levels, providing financial assistance and social support services can help mitigate the impact of financial strain on mental health. Additionally, policies should focus on the specific stressors faced by young people and women, as these were the identified groups with higher EPD.

### Strengths and limitations of the study

A major strength of this study was the utilisation of two high-quality, nationally representative data sources that were collected by the ABS and the Australian Government Department of Social Services, with the aim of informing health and social policies in Australia. Moreover, this study used a matched comparison group and advanced statistical approaches, and the matching methodology is novel (building on the methodology derived by JE (Shawyer *et al.*, 2014) and provides an exemplar for other researchers to extract policy information from existing large data assets. Our study addressed the existing inconsistencies in the prevalence of EPD between humanitarian migrants and the host population using a large sample of study participants.

Despite these strengths, it has a few limitations. Although the response rates for both surveys were within acceptable ranges for large-scale population surveys, non-response bias remains a potential concern. This may limit the generalisability of the findings and could lead to underestimation or overestimation of the true prevalence of EPD. Future studies should consider implementing strategies to adjust for non-response bias. The reliance on self-report data more generally can also impact comparability since self-rated health may be influenced by cultural norms and individual perceptions. The final limitations relate to the K6, which was used instead of the K10 resulting in a potential loss of some information and a more constrained screening of mental illness (Furukawa *et al.*, 2003). There was also no confirmation of clinical diagnosis so the use of K6 alone as a screening tool has the potential to result in misclassification and inflate prevalence estimates (Shawyer *et al.*, 2017). Finally, this study employed a categorical cut-off (K6 ≥ 19) to define EPD, which is a common method but may limit sensitivity to variation in distress levels. Categorical thresholds can be somewhat arbitrary and may obscure more subtle gradients in mental health conditions. Future studies could benefit from examining continuous K6 scores to enhance the robustness of findings and better capture the full range of psychological distress.

## Conclusion

This study found that EPD was more prevalent among humanitarian migrants compared to the Australian-born population, and this difference increased when self-rated health was considered. These results highlight the need to prioritise both physical and mental health interventions for this group. The findings point to significant disparities in EPD related to education levels, suggesting that these factors play a critical role in the mental health of both groups. Interestingly, income disparities were a prominent factor in EPD for the Australian-born sample, but not the humanitarian migrants. Overall, these findings indicate the necessity of routine screening for EPD among humanitarian migrants, as early identification can help prevent the progression to more serious mental disorders. Moreover, early and culturally appropriate screening, diagnosis and intervention are not only more effective but also more cost-efficient than addressing mental health complications at the resettlement phase of migrants, minimising long-term healthcare and social expenses.

## Data Availability

Data from the Building a New Life in Australia (BNLA) study are available to authorised users through the National Centre for Longitudinal Data, and data from the Australian National Health Survey (ANHS) are accessible via the Australian Bureau of Statistics website.
